# Metabarcoding provides accurate estimation of volumetric diet composition in a top predator despite interference from blocking primers

**DOI:** 10.1038/s41598-025-14837-9

**Published:** 2025-08-08

**Authors:** Jabi Zabala, Pablo Acebes, María J. Madeira, Efrén Fernández, Benjamín Juan Gómez-Moliner, Xabier Cabodevilla

**Affiliations:** 1 Department of Zoology and Animal Cell Biology, University of the Basque Country UPV/EHU, C/Paseo de la Universidad 7, Basque Country, 01006 Vitoria-Gasteiz, Álava-Araba Spain; 2 Terrestrial Ecology Group (TEG-UAM), Department of Ecology, Universidad Autónoma de Madrid, 28049 Madrid, Spain; 3 Centro de Investigación en Biodiversidad y Cambio Global (CIBC-UAM), Universidad Autónoma de Madrid, 28049 Madrid, Spain; 4 Sendaviva, Parque de la Naturaleza de Navarra, S/A. Ctra. Virgen del Yugo s/n, 31513 Arguedas, Navarra Spain; 5 Conservation Biology Group, Landscape Dynamics and Biodiversity Program, Forest Science and Technology Centre of Catalonia (CTFC), Solsona, Spain

**Keywords:** Carnivore, Wolf, *Canis lupus*, Diet quantification, Environmental DNA, Feces, Ecology, Ecological genetics

## Abstract

The study of diet is central to wildlife ecology, management and conservation. Metabarcoding enhanced the ability to identify species contributing to wildlife diets, and blocking primers can maximize prey detection. Relative read abundance (RRA) of different prey species has been used as semi-quantitative approach, assuming that RRA reflects species’ contributions to diet. We tested accuracy of RRA in diet estimation by feeding captive wolves six different diets. We analyzed samples both without and with four blocking primer concentrations (5, 10, 15 and 20×). RRA provided a highly accurate representation of the overall dietary composition, with best results obtained without blocking primer (0.775 ± 0.033; *P* < 0.001; *R*^2^ = 0.815). While the use of blocking the primer resulted in higher proportions of reads for diet items it did not increase the probability of detecting diet components. Moreover, the blocking primer led to increased detections of items not fed to wolves and produced slightly less accurate estimates of diet composition. Resampling indicated that sample sizes beyond 30 scats improved the accuracy of diet estimation. Our results are promising and support the use of metabarcoding to determine volumetric diet but caution and further research are necessary before extrapolating findings.

**Supplementary Information:**

The online version contains supplementary material available at 10.1038/s41598-025-14837-9.

## Introduction

The study of diet has been central to ecological sciences since their origin. Accurate knowledge of animal diets is crucial in many research areas of wildlife management, ecology and evolution, including: community structure and species coexistence through trophic niche partitioning^1,2^; wildlife management and conservation^3^; human-wildlife conflict management and mitigation^4^; determination of trophic webs and their structure^5^; quantification of ecosystem services provided by animals^6^; and various aspects of animal autecology such as abundance, distribution, movement, time allocation or breeding success^7^.

Not surprisingly, given its relevance, the study of diet has received considerable attention from researchers, and several methods have been developed^8,9^. Trophic research studies include isotope analysis to ascertain trophic position within the community^10^ the tracing of biomolecules across food webs^11^ and the description of diet through the identification of prey remains. The latter is, perhaps, the most commonly employed method, typically involving morphological or molecular identification of prey from feces, other excreta, or other non-destructive sampling method. Methods used to describe diet can be classified into two main groups: frequency of occurrence methods, which determine how often, or in how many samples, a particular food item appears, thus profiling the diet; and volumetric methods that aim to determine the relative contribution of each food item to the overall diet^12–14^. These methods are complementary and provide different insights into animal diet. When combined, they can offer a comprehensive perspective with detailed information on dietary composition.

Both approach types, frequency of occurrence and volumetric methods, depend on researchers’ capability to correctly identify prey remains. Initially, prey identification relied on morphological analysis of remains, imposing strong limitations on diet research. The development of molecular techniques brought about a major breakthrough in the capability to identify prey remains. In particular the technique of high-throughput sequencing in combination with DNA barcoding across multiple taxa in a mixed sample (DNA metabarcoding hereafter^8,15,16^. Indeed, studies comparing results of DNA metabarcoding and morphological analyses of remains in feces reported that DNA metabarcoding achieved greater taxonomic resolution and identified more rare food items than morphological methods^17–19^. Further, early researchers suggested that DNA metabarcoding could be quantitative, assuming that the proportion of reads (relative read abundance) or number of sequences obtained of each species from each sample correlate with the biomass of the species in the original sample or in the food represented in the fecal sample^20^. However, this quantitative approach has been strongly contested^8,20–22^ and several critical steps have been pointed out. For instance, different tissues may contain different amounts of DNA^16^ and different tissues or species may be harder to digest^23^. In addition, primers used in amplification might favor some species over others^24^ and there might be issues associated with sequencing machines used, data analysis pipelines and others (issues reviewed in detail in Ando et al., 2020; Hoenig et al., 2022; Lamb et al., 2019; Liu et al., 2021; Nielsen et al., 2018). A review and meta-analysis of the quantitative approach reported a weak relationship between biomass and the relative read abundance in the sample, yet with a large degree of uncertainty^20^. Several empirical experiments have been conducted to assess the quantitative accuracy of DNA metabarcoding using mock communities composed of known mixtures of specimens^25^. However, despite the pressing need for them^8^ few attempts have been made to analyze the quantitative reliability of DNA metabarcoding for diet assessment using fecal samples from animals with known diets. Experimental studies so far are scarce, having fed a few species in constant proportions and reported mixed results regarding the quantitative approach^23,26–28^ possibly influenced by the species included in the diet, the primer selected, and other factors^22,28^.

An issue specific to diet study using DNA metabarcoding is that samples are rich in consumer/predator DNA^25,29^ which has not undergone a digestive process and is better preserved than prey DNA. As a consequence, when predator and target prey are phylogenetically close and are amplified by the same primer, most of the sequence reads obtained through DNA metabarcoding are of the consumer/predator species, with reads of prey items being less, or sparsely, represented. To overcome that, researchers use blocking primers^25,30^; a primer which specifically targets predator DNA and prevents its replication^31^. However, few studies have assessed the influence of blocking primers on relative read abundance of consumed items and results are contradictory^32^. For instance, a study with spiders found that blocking primers significantly altered relative abundance of reads from a mock arthropod community^25^. In contrast, a study with raccoon dogs (*Nyctereutes procyonoides*) found no significant impact of blocking primer on the mean relative abundance of reads of prey species^33^. Further, blocking primer can be used at different concentrations, and it is unclear if that might influence results as well. Thus, the influence of the use and concentration of blocking primer in relative abundance of prey items, which is key for the quantitative approach, remains largely unassessed.

Finally, the window of time represented in each sample –i.e. food items taken over how many days are represented in each sample– is a question common to all diet study methods, whether morphological or molecular, and is of considerable ecological and management relevance. Controlled trials with predators suggest periods of time ranging from two to four days or up to 60 days^26,27,34^. Although this issue might not be important in terms of assessing the quantitative performance of DNA metabarcoding, it is very relevant to frame the results within an ecological perspective. This helps to place the results within the behavioral ecology and activity and movement patterns of the species.

To answer or provide insight into (1) the validity of the semi-quantitative DNA metabarcoding approach, (2) the influence of blocking primer on results, and (3) the window of time represented in each scat, we fed captive wolves six diets composed of three to seven prey species in different proportions, using nine different prey species. We fed wolves the same diet for a week with food offered each day, and included a small amount of other seven species used as time markers. We collected wolf scats and used DNA metabarcoding without and with blocking primer at four different concentrations to assess its performance as a quantitative estimator of diet. We used time markers to approximate the time window sampled in each scat and to test whether blocking primer increased the power to detect rare items.

## Results

### DNA extraction and sequencing

In a first analysis conducted without blocking primer, the two extraction blanks produced 89 and 149 reads, all of which belonged to wolf DNA. The PCR blank of that analysis produced 15 reads, again all of them wolf sequences, suggesting an absence of contamination. In this run we obtained a total coverage of between 2 694 and 93 670 reads per sample, resulting in a dietary DNA coverage ranging from 156 to 13 500 reads per sample. In a second analysis conducted with blocking primer the same two extraction blanks with 10× blocking primer added resulted in 93 and 185 reads, and again all of them were wolf sequences. The PCR blank, in turn, yielded 413 reads, 301 of which corresponded to wolf and the remaining 112 to turkey. However, a thorough inspection of the results did not provide evidenced of general contamination with turkey DNA in the samples. In this case, we obtained a coverage for dietary DNA of between 200 and 133 357 reads per sample (mean = 28 062 reads) with 5× blocking concentration; between 252 and 76 932 reads per sample (mean = 20 221 reads) with 10× blocking concentration; between 575 and 121 037 reads per sample (mean = 22 469 reads) with 15× blocking concentration; and between 301 and 98 904 reads per sample (mean = 17 369 reads) with 20× blocking concentration. The analysis of mock samples showed no evidence of considerable extraction or primer bias (Sup Mat; Table S2 and Fig. S1).

### Effect of blocking primer on relative read abundance of diet items

**Fig. 1:**
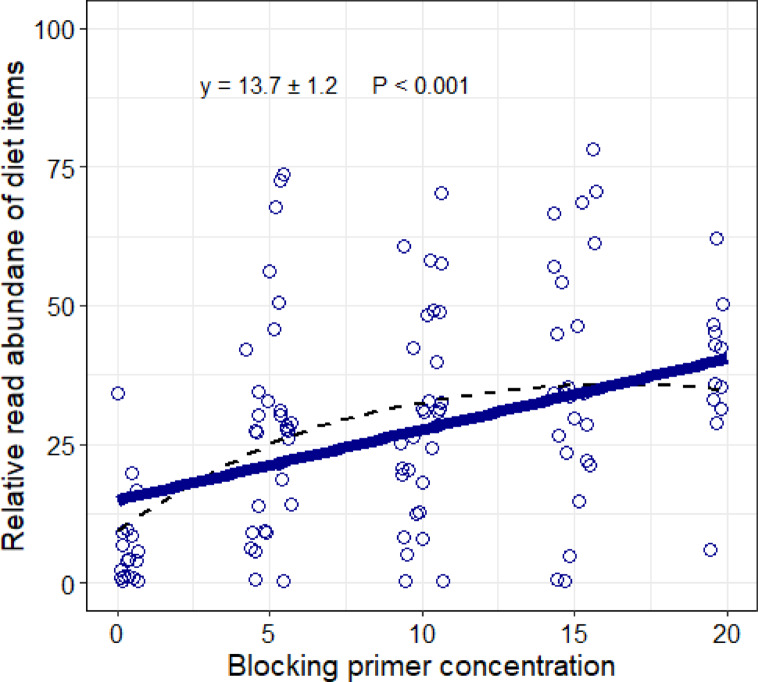
Proportion of reads assigned to diet items in relation to blocking primer concentration. Points represent the proportion of diet reads in scats and have been slightly jittered horizontally to enhance visibility. The solid blue line shows the linear GLM fit to the data, while the broken thin line represents the smoothed trend, showing a marked increase in the proportion of diet reads with increasing blocking primer concentrations, which becomes nearly flat beyond 10× blocking primer concentrations.

The use of blocking primer significantly increased the representation of diet items in reads (y = 13.66 ± 1.21 x blocking primer concentration; Fig. 1). The percentage of diet reads increased from 5.58% (± 8.23) without blocking primer to 34.45% (± 18.09) with x20 blocking primer concentration. However, a smoothed line fitted to the results suggested a threshold effect, with a marked increase in diet reads with 5× blocking concentration and smaller increases with higher concentrations of blocking primer (Fig. 1).

### Numbers of items fed detected and effect of blocking primer

**Fig. 2:**
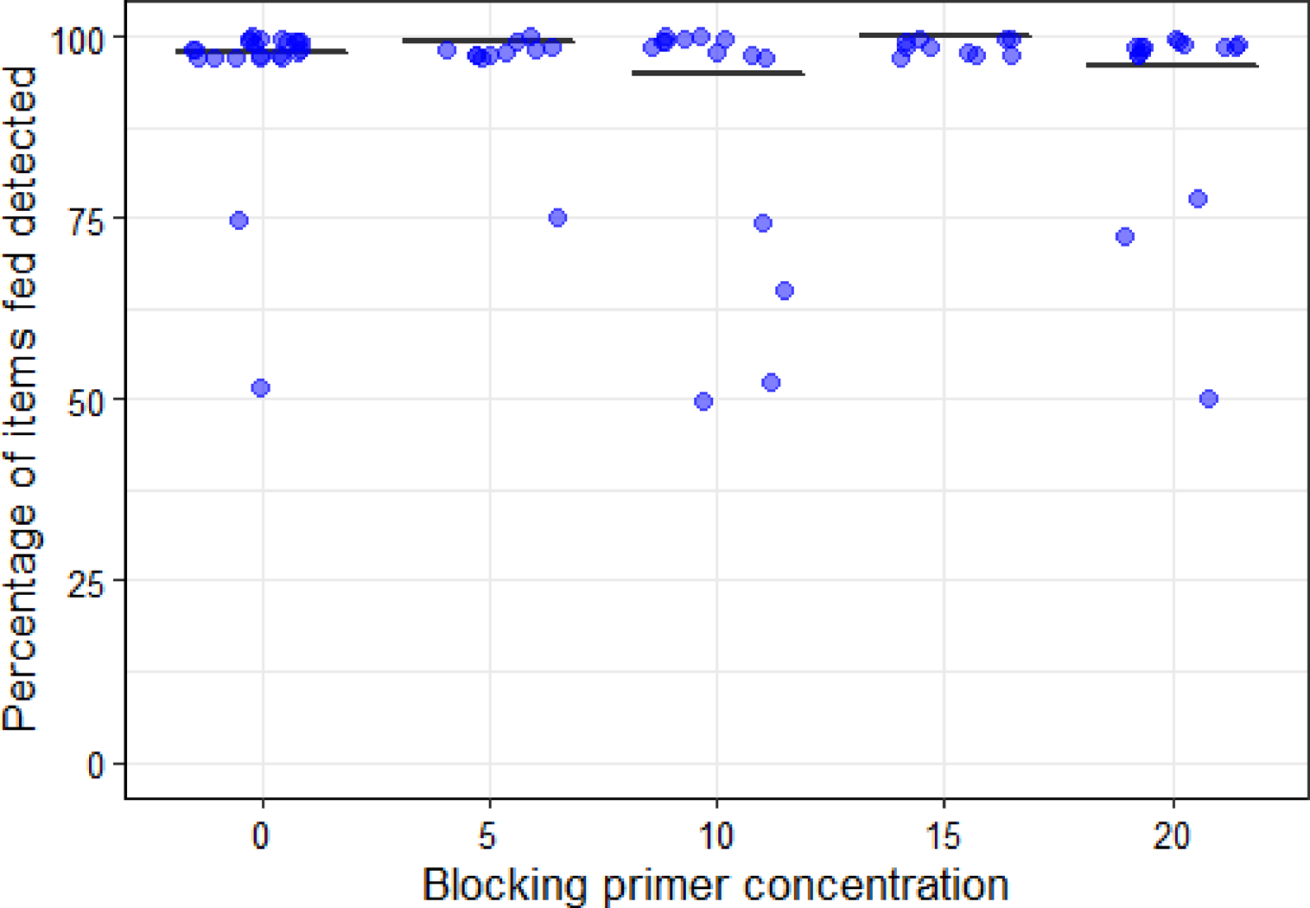
Percentage of fed items detected in each sample at different blocking primer concentrations. Points represent the percentage of items fed detected in each sample and the black solid lines indicate the average value predicted by the GLM (Table 1). Points have been slightly jittered vertically (± 3%) and horizontally to enhance visibility.

The probability of a diet item fed being detected in the results was not associated with the use or the concentration of a blocking primer (Table 1). Of the 136 samples included, considering the same scat with different blocking primer concentrations as different samples, only in 10 samples, involving seven different scats, were some of the food items not detected: in four cases, two of four items were detected, in one case, two out of three were detected, and in the other five cases, only three out of four items were detected (Fig. 2). Items were missed in two out of 34 samples without blocking primer, in one out of 30 samples with 5x blocking concentration, in four out of 30 samples with 10× blocking concentration, and in three out of 25 samples with 20× blocking concentration. In total, 14 out of 1768 items were missed and, on average, missed items composed 19.28% (± 21.83; range 5–70%) of the diet offered.

**Table 1:** Results of the proportional GLM assessing differences in the probability of an item in the diet being detected based on the concentration of the blocking primer as a categorical factor. For each blocking primer concentration used, with the intercept being the no blocking primer condition, we show the regression estimate (Est) and its standard error (SE), the value of the z statistic (z val), and the associated P value (*P*). We also show the average proportion of fed items detected per sample (% items detected) with its standard deviation in parentheses and the sample size (N).

Blocking primer concentration	Est	SE	z val	*P*	% items detected	*N*
Intercept (0×)	3.836	0.584	6.573	< 0.001	97.8 (9.5)	34
5×	0.935	1.162	0.805	0.421	99.1 (4.7)	28
10×	-0.900	0.718	-1.253	0.210	94.3 (14.7)	28
15×	16.576	1649.287	0.01	0.992	100 (0.)	23
20×	-0.668	0.775	-0.862	0.389	95.7 (12.3)	23

### Time window represented in scats and blocking primer effect

**Fig. 3:**
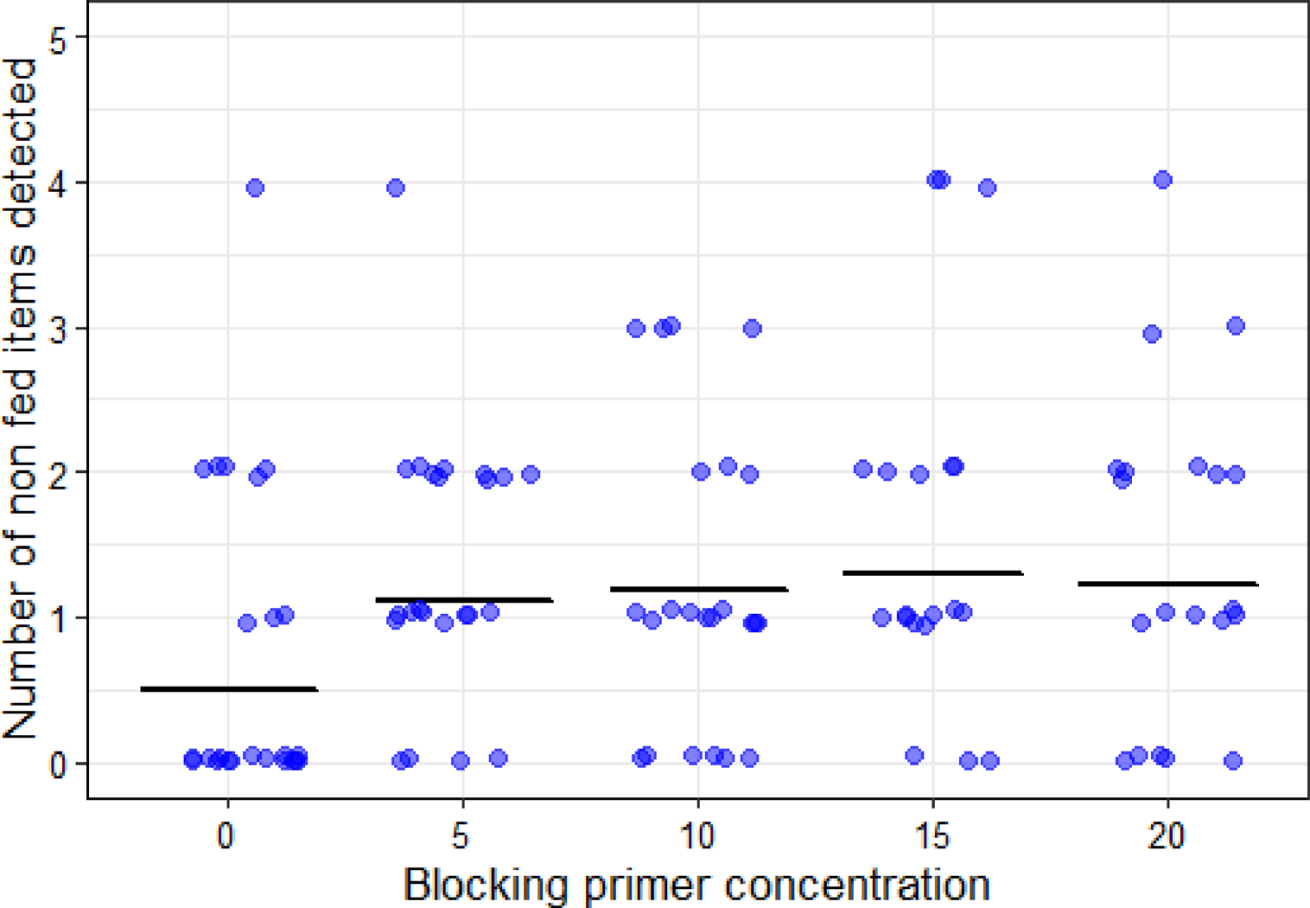
Number of wrong items (i.e., items not fed in the diet or markers not yet fed) in each sample at different blocking primer concentrations. Points represent the number of wrong items detected in each sample, and the black solid lines indicate the average value predicted by the GLM (Table 2). Points have been slightly jittered vertically (± 0.05) and horizontally to enhance visibility.

The use of blocking primer tended to increase the number of daily markers detected. This difference was statistically significant with 5× blocking primer concentration, nearly significant with 10× and 15× blocking primer concentrations, and non-significant with 20× blocking primer concentration (Table 2). However, the blocking primer did not increase the time window represented in samples (blocking primer concentration; 0.013 ± 0.012; t value = 1.023, *P* = 0.31; *N* = 78). On average, without blocking primer we detected markers fed 1.61 (± 0.12) days before the scat was collected. Considering all samples, with and without blocking primer, the time window was 1.73 (± 0.08) days (Table 2). On the other hand, the addition of blocking primer also enhanced the detection of wrong items, items that were not fed to the wolves (Table S3). Wrong items appeared with all blocking concentrations but more than doubled from 0.5 per scat when no blocking primer was added to more than one with any blocking primer concentration (Fig. 3). However, wrong items appeared with low numbers of reads (1.6 ± 4.3% of diet reads in sample), comparable to species used as time markers (2.0 ± 2.6% of diet reads) and less than food items contributing at least 5% of the diet mass (5.3 ± 4.7% of reads).

**Table 2:** Results of the negative binomial GLM assessing differences in the number of markers detected based on the concentration of the blocking primer, treated as a categorical factor. For each blocking primer concentration used, with the intercept being no blocking primer condition, we show the regression estimate (Est) and its standard error (SE), the value of the t statistic (z val), and the associated P value (*P*). We also show the average time window, the average maximum number of days represented by markers in each sample, with its standard deviation in parentheses and the sample size (N), with the number of samples in which at least one marker was detected in parentheses.

Blocking primer concentration	Est	SE	z val	*P*	Time window	*N*
Intercept (0×)	-0.961	0.277	-3.466	< 0.001	1.50 (± 0.67)	34 (12)
5×	0.925	0.338	2.740	0.006	1.80 (± 0.83)	28 (20)
10×	0.674	0.353	1.909	0.056	1.71 (± 0.69)	28 (17)
15×	0.659	0.368	1.789	0.074	1.86 (± 0.77)	23 (14)
20×	0.599	0.373	1.603	0.109	1.79 (± 0.70)	23 (14)

### Accuracy of the DNA metabarcoding approach and effect of the blocking primer

**Fig. 4:**
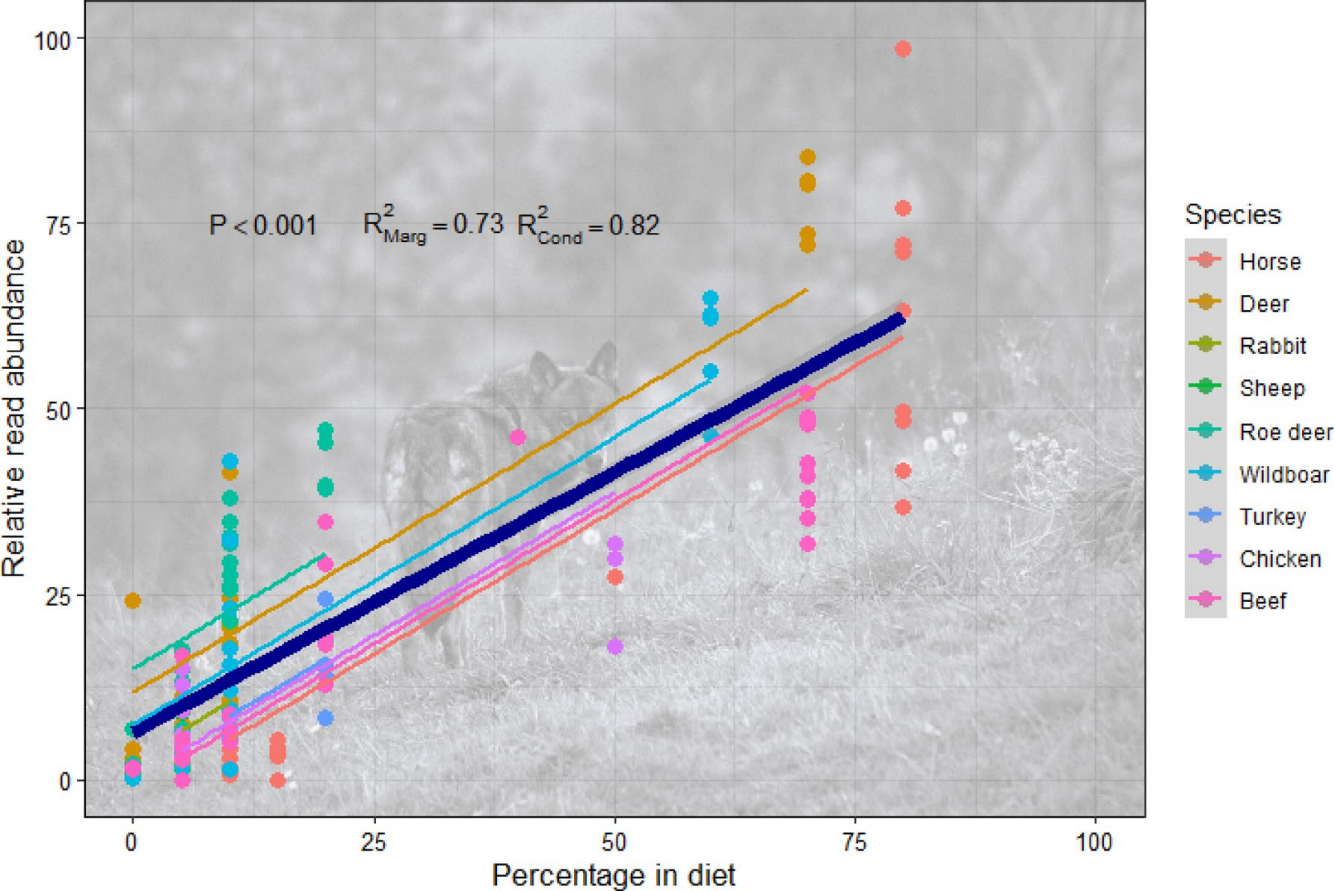
Association between the composition of fed diet and the relative read abundance (RRA) without a blocking primer. Points represent the proportion of each diet item in samples. The thick blue line indicates the average trend produced by the GLMM with diet species as random effect. Thinner lines show linear regressions fitted over predicted values for different diet species included in the study.

Semi-quantitative results of metabarcoding yielded a highly accurate representation of the diet fed (Fig. 4). The model without blocking primer accounted for 81.5% of the variation in the data, and most of that (73.4%) was accounted for by fixed factors (i.e. by the relative proportion of items fed; while diet species, as random factor, accounted for a further 8.1%. This was shown by the GLMM with percentage of each item in diet as response, proportion as predictor, and diet species as random factor (Table 3). Adding scat identity as crossed random to the same model yielded the same results and it accounted for no variance in the intercept at all. Thus, we discarded scat identity in this and subsequent models. Results obtained using a blocking primer also produced an accurate depiction of the diet fed (Table 3; Figs. S2, S3, S4 and S5). However, adding a blocking primer resulted in lower coefficients of determination in every case, without a clear linear association between blocking primer concentration and the reduction in the amount of explained variance (Table 3). In other words, the use of blocking a blocking primer slightly weakened the association between the diet fed and RRA. Furthermore, the addition of a blocking primer increased the amount and proportion of the coefficient of determination attributable to random factors. That is, the amount of variation explained by diet species rather than by the amount of species eaten. This was shown by the increasing difference between marginal and conditional *R*^2^ with increasing blocking primer concentrations That difference was 0.081 when no blocking primer was used, representing 9.94% of the total variance explained (*R*^2^_*con*_ = 0.815). With increasing blocking concentrations, the difference between *R*^2^_*marg*_ and *R*^2^_*con*_ was 0.139 (19.33% of the total variance explained), 0.185 (28.20%), 0.149 (19.35%) and 0.158 (22.83%) for 5×, 10×, 15× and 20× blocking primer concentrations, respectively. Thus, the addition of the blocking primer did not only reduce the coefficient of determination but also doubled the contribution of the random factor to the total *R*^2^. That is, the actual diet eaten lost importance in the *R*^2^ of the regression, while the over- and underrepresentation associated with meat type became more relevant. This is evident in the greater dispersion of individual regressions for each diet species around the regression line in the regressions obtained using a blocking primer (Figs. S2, S3, S4 and S5) compared to the regression without a blocking primer (Fig. 4).

**Table 3:** Results of the GLMM analyzing the association between the fed diet composition and the semi-quantitative metabarcoding approach for each blocking primer concentration assessed. For random variables we show their variance (Var) and the standard error (SE) associated with them. For fixed factors we show the estimate (Est) of the slope and its standard error (SE), the value of the t statistic (t val), and the associated P value (*P*). We also indicate the sample size (N) in each model and its marginal (*R*^2^_*marg*_) and conditional (*R*^2^_*con*_) coefficient of determination.

Blocking primer	Variable						
	Fixed	Random	Var	Est	SE	t val	*P*
0×							
*N* = 158		Species (*N* = 9)	43.48		6.594		
*R*^*2*^_*marg*_ = 0.734	Intercept			4.019	2.476	1.623	0.136
*R*^*2*^_*cond*_ = 0.815	Diet			0.775	0.033	23.45	< 0.001
5×							
*N* = 160		Species (*N* = 9)	82.41		9.078		
*R*^*2*^_*marg*_ = 0.580	Intercept			4.484	3.331	1.346	0.209
*R*^*2*^_*cond*_ = 0.719	Diet			0.765	0.045	16.91	< 0.001
10×							
*N* = 163		Species (*N* = 9)	116.2		10.78		
*R*^*2*^_*marg*_ = 0.471	Intercept			4.919	3.903	1.26	0.238
*R*^*2*^_*cond*_ = 0.656	Diet			0.711	0.052	13.61	< 0.001
15×							
*N* = 137		Species (*N* = 9)	93.47		9.668		
*R*^*2*^_*marg*_ = 0.621	Intercept			3.081	3.503	0.879	0.401
*R*^*2*^_*cond*_ = 0.770	Diet			0.838	0.047	17.827	< 0.001
20×							
*N* = 134		Species (*N* = 9)	101		10.05		
*R*^*2*^_*marg*_ = 0.534	Intercept			3.842	3.721	1.033	0.326
*R*^*2*^_*cond*_ = 0.692	Diet			0.781	0.055	14.131	< 0.001

### Sample size and accuracy of DNA metabarcoding

**Fig. 5:**
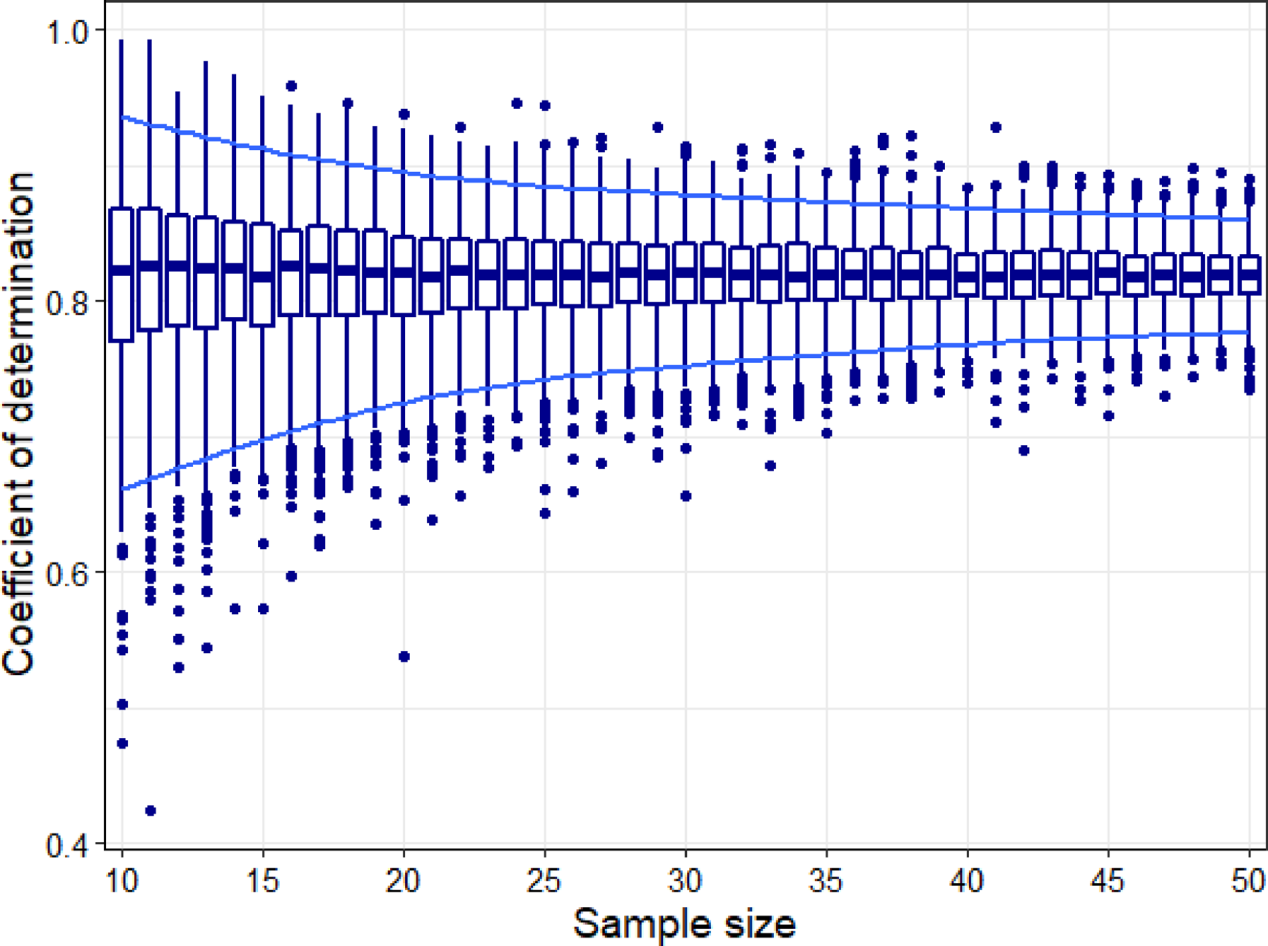
Resampling results showing variation in the conditional coefficient of determination (*R*^2^_*con*_) with sample size. Boxplots show the distribution of *R*^2^_*con*_ based on 1000 resamples for each sample size. The box encompasses values within the 75th and 25th percentiles, the thick line indicates the median value, and the thin vertical lines show the range of values after discarding outliers (solid dots). The blue horizontal lines show the 95% confidence intervals.

Finally, the resampling analysis showed a clear increase in the precision of diet estimation with sample sizes stabilizing somewhere around 35 scats (Fig. 5). That is, estimations of diet based on small sample-sizes showed significant variation around the mean. That variation decreased with increasing sample size, and although the decrease never stopped, the per-unit improvement became nearly negligible beyond 35–40 samples.

## Discussion

Our results showed that the metabarcoding approach offered a highly accurate depiction of the composition of diets fed, with relative read abundance (RRA) indicating the actual proportional mass contribution. Furthermore, the metabarcoding approach was most accurate without a blocking primer, although the coefficient of determination of the regression remained high across all blocking primer concentrations. Using a blocking primer increased the proportion of reads corresponding to diet items and the probability of detecting trace food items, such as our time markers, but there were no differences for items that composed at least 5% of the diet. On the other hand, the use of a blocking primer increased the probability of obtaining reads of items that were not part of the fed diet and, as stated, slightly weakened the association between diet composition and RRA.

Our metabarcoding results showed a considerably better fit than previous approaches^20,26,35^. Lamb et al. (2019) conducted a meta-analysis using the slope of the association between biomass and sequences obtained to assess the quantitative relationship and obtained an average slope 0.52 ± 0.34. Slopes in our models ranged between 0.71 and 0.84 (Table 3), all within the high end of the range found in the meta-analysis. This good performance is particularly relevant because only one of the 22 studies included in meta-analyses used fecal material as starting material while most others started with biomass or DNA. Thus, regarding diet analysis, our study is more realistic than most of the previous ones as it included further steps that could lead to relevant biases^20^. Previous studies using fecal material as source fed a constant mixture of three fish species and analyzed sequences recovered from fecal material^26,28^. Several already reported issues could cause the differences between their results and ours, including differential species digestibility and sequencing issues^20^. A recent review on diet analysis techniques criticized the extremely limited diversity of prey items used in experimental assessment of metabarcoding so far, and suggested that direct comparisons might be ineffective for complex dietary mixtures^8^. Our study included more species than any previous study (nine vertebrate species in 6 different diets), and obtained a better fit between diet and numbers of sequences. However, we acknowledge it is still a limited number of prey species. If species-associated errors –e.g. differences in digestibility, amounts of DNA, primer affinities– vary randomly among species, including more species could control for biases and result in an, overall, better approximation to real diet. In addition our results showed that approaches with low sample sizes are prone to substantial bias (Fig. 5). For instance, the penguin study only included three meta-samples from a constant mixture of three species^26^. Our results suggest they might have obtained a higher fit if they had included more replicates. Previous studies differentiated between within-species and across-species quantification. Within-species quantification refers to the estimation of the abundance of a certain item across different samples, while across-species quantification refers to the estimation of the contribution of each species to a single sample^36,37^. Our study was not designed to assess across-species quantification. However, although the high coefficient of determination suggests a good approximation for large samples, the variation associated with species as random factors and the considerable error associated with some individual values (Fig. 4) suggest that it might not be a safe approach when accurate values for each sample are required. In such cases, other approaches, such as using shotgun sequencing and correction factors might provide more accurate quantifications^36,37^. Indeed, our resampling analyses showed that dietary analyses based on sample sizes smaller than 30 might introduce considerable error in dietary estimation.

The effects and consequences of using blocking primers have been scarcely tested and with contradictory results^32^. In our case, the addition of a blocking primer reduced the association between the proportion of biomass fed and the sequences obtained. However, it still showed a good fit, in accordance with studies suggesting no significant impact of blocking primer in the relative abundance of reads obtained^33^. The results of our mock samples suggest a slightly higher primer affinity for Cervidae (deer and roe deer), while slightly underrepresenting chicken, beef, and horse (Fig. S1). As we found no difference between mock samples created from biomass or DNA mixtures, our results suggest no significant differences in DNA contents among species or extraction bias and differences likely arise from differences in the primer affinity. Diet results seemed to increase the bias associated with primer affinity (Fig. 4), while the addition of blocking primer further exacerbated them (Figs. S2, S3, S4 and S5). We currently lack an explanation for the observed species-specific bias in diet analysis without the blocking primer. However, its increase with the blocking primer could arise from the quadratic nature of DNA replication in the PCR and augmented differences in the amount of sequences of each species in each replication cycle. The addition of the blocking primer increased the amount of trace items detected too, which agrees with common results of blocking primers enhancing reads and detection of target species^32,34,38^. However, the addition of blocking primer also produced more sequences that were not part of the diet fed. In every case, the items detected were present in some of the other diets or were day markers not yet fed in that week. It might be that the addition of a blocking primer detected trace items fed several days before, but it more likely increased sequences attributable to small contamination either in the field or in the laboratory. Although we cannot ascertain the source of those errors, it calls for caution. The total numbers of reads of wrong items were in general very small and bias could be prevented by deleting items below a certain threshold of reads. Yet, in our case, that would have resulted in deleting some real food items too. The amplification of DNA unrelated to the sample is something to be carefully considered, particularly in studies that analyze data based on frequency of occurrence.

The daily markers detected suggested, on average, a time window of 1.5 days of food. This is consistent with research reporting that species detection was highest for food items consumed one day prior to scat collection^34^but shorter than the at least four days reported for penguins^26^. This is also consistent with previous research reporting gut passage time of about two days in canids of 14 to 57 h^39,40^. However, we provided daily markers in very small amounts, and previous research has shown that the probability of detecting items increases with the proportion of prey consumed^34^. Thus, it is likely that DNA of prey consumed in large amounts will persist longer in the feces, although likely in small amounts. How consumption of different prey in consecutive days influences diet representation in metabarcoding analyses remains an open question.

Overall, our results suggest that, at least for carnivores and under our experimental settings, metabarcoding offers an accurate reflection of global diet consumed. Particularly, if 30 or more samples are available for each season or unit for which we want to determine the diet (area, pack, period of time…). Our results also discourage the use of blocking primers unless the goal of the study includes determining items eaten seldom or in very low quantities. In any case, studies using a blocking primer should be particularly cautious with contamination and critical in the analysis of sequences, as our results suggest that blocking primers might render the process more susceptible to contamination. Although our results are very promising and suggest that metabarcoding can yield an accurate depiction of diet, there are several aspects that call for caution and need to be carefully considered before extrapolating our results to wildlife diet studies.

In addition to many others, there are four main ***caveats*** we need to raise. Firstly, our experimental diets were unrealistic as wildlife will hardly eat the same species in the exact same proportions for several consecutive days. If a large number of samples are collected randomly over time, results should converge towards the real contribution of each time to diet. Yet, how long a species consumed in a considerable amount and how its RRA will decrease over time remains unknown and can deeply impact results and bias inference from the results. Secondly, while our diets only included lean meat in an attempt to reduce differences in DNA concentration and tissue digestibility^16,23^ wild animals often eat other parts like skin, hair and bones that are likely to impact the results. Similarly, fatty tissues can provide a relevant amount of energy but comparatively less DNA. Thirdly, we fed our wolves every day, while wildlife feeding can be irregular over time, particularly for predators. Wild animals often experience periods of gorging on prey, intercalated with days of starvation. Food abundance or paucity might affect digestion efficiency, scat abundance, and DNA content, thus affecting diet estimation. Lastly, all our diet species were vertebrates, and we used a single primer pair. Diets of animals that include items that are phylogenetically diverse and require the use of more than one primer might show higher primer affinity biases and further biases associated with differential primer performance. Similarly, other primers used to study diet might perform differently.

## Methods

### Study species, location and feeding trial

We used Iberian wolves (*Canis lupus signatus*) as the study species. We selected wolves for several reasons. Their diet has been widely studied and is still a common and relevant study subject. In addition, numerous studies have analyzed wolf diet using both traditional and molecular methods with and without blocking primer, including cases of conservation, management, wildlife conflict, trophic niche width and shift, as well as intraguild predation and control^4,29,41–44^. Finally, wolves are primarily carnivorous preying mainly on large and medium-sized mammals, and occasionally on small mammals and birds. Invertebrates, fruits and other vegetable materials may appear with some frequency but usually are not relevant in volumetric terms^45^. Thus, a realistic study of diet could be performed using a single primer targeting vertebrate DNA.

We conducted the study in Sendaviva, Parque de la Naturaleza de Navarra, during the summer of 2022. Sendaviva is a zoo in Arguedas (Navarre, Spain) that, at the time, housed a pack of seven Iberian wolves within a 0.325 hectare enclosure with forest and open areas. For feeding trials we used two healthy wolves that were temporarily housed in facilities used for the observation and monitoring of sick animals, the acclimation of new individuals to the pack and similar uses. The facilities included separate indoor and small outdoor areas, and a perimeter fence in the open areas that prevented the entry of any wildlife larger than mice. In collaboration with Sendaviva personnel we prepared a feeding and sampling protocol and conducted a training session with wolf zookeepers. All feeding and scat collection were performed by Sendaviva zookeepers. ARRIVE guidelines were followed during the study. All procedures related to wolf housing, feeding and scat collection were performed in accordance with relevant guidelines and regulation and an ethics approval was deemed unnecessary according to national legislation^46^. Sendaviva is a registered Zoo (ES3103000700) licensed to house wolves, none of the procedures in the study departed from regular wolf caretaking activities and the study design was reviewed and approved by its animal welfare department.

### Diet composition, deeding trial and scat collection

We used meat of nine vertebrate species to create six diets with different prey composition (Table 4). Meats used included beef (*Bos taurus*), horse (*Equus ferus caballus*), wildboar / pork (*Sus scrofa / S. scrofa domestica*), deer (*Cervus elaphus*), roe deer (*Capreolus capreolus*), sheep (*Ovis aries*), rabbit (*Oryctolagus cuniculus*), turkey (*Meleagris gallopavo domesticus*), and chicken (*Gallus gallus domesticus*). To select species included in diets, we prioritized vertebrate species commonly found in the food of wild wolves^47–49^. However, despite birds being uncommon in wolf diets, we included two avian species widely available to complete the diets and increase the number of levels for the random factor^50^. We obtained all meat form markets, deer culling programs, and, occasionally, from local hunters. All the meat used in the experiment fulfilled sanitary standards for human consumption. As different tissues may have different amounts of DNA^16^ we only used lean meat to reduce possible biases associated with digestibility, fat or water content, and other factors. We combined meats in diets in different proportions, from 5 to 80%, to generate samples with a wide range of relative volumetric contribution and to ensure representation across different areas of the regression of actual to estimated diet composition (see Statistical Analyses).

**Table 4:** Composition of the diets used in this study. We show the proportional contribution of each meat type to each diet as a percentage.

Diet	Diet 1	Diet 2	Diet 3	Diet 4	Diet 5	Diet 6
Beef	70	5	10	20		40
Horse	10	80	15			50
Deer	10		70		5	
Roe deer	10		5		20	
Wildboar/pork		10		60	5	
Sheep					5	
Rabbit					5	10
Turkey				20	10	
Chicken		5			50	

We fed the wolves each of the six diets for seven consecutive days (Table 4). To ensure that actual food intake by wolves’ accurately matched diet composition, we presented their food in 100 g meatballs. We chose 100 g meatballs because it is less than wolves can consume in a single mouthful^51^ ensuring that they ate food items in the right proportion regardless of the their total food intake. Therefore, we minced all the meat types, weighed them to the nearest gram and mixed them into 100 g meatballs, ensuring that each meatball contained the same proportion of meat types as the corresponding diet. We processed all the meat in a food processing room with freezers and several workstations. To prevent cross-contamination, we minced meats separately using an electric meat shredder. Before and after mincing each meat type, we thoroughly washed the shredder and its parts with a 50% bleach solution and allowed them to air dry. We also washed all workstations, knives and other materials used with a 50% bleach solution. We prepared each diet in separate work stations. We also included a daily marker in meatballs. Markers consisted of small amounts (< 1 g) of meat from seven species other than those included in the diets (table S1). We included markers to gains insight into the window of time represented in scats. Once prepared and mixed, we individually wrapped each meatball with plastic foil and stored them frozen. We prepared 20 meatballs for each day of the experiment (140 per diet) and organized them by diet type and day to facilitate the feeding process.

The feeding trial lasted 42 days, from May 28 to July 11, 2022. Two day prior to the start, we moved the meatballs of the first day from the freezer to the refrigerator to allow them to thaw. Each morning, we fed the wolves the corresponding 20 meatballs and moved those for two days later to the refrigerator. We offered the meatballs in the indoor areas and momentarily locked the wolves in there while caretakers cleaned and inspected the outdoor areas for scats. The wolves accepted the meatballs without issue. Later, when the wolves were outside, zookeepers cleaned the indoor areas and inspected them for scats. We discarded scats from the first three days of each diet to avoid possible presence of food items taken before the start of the diet^26^ and, thus, unrelated to the experiment. From the fourth to the seventh day of each diet, we collected scats for later analysis. In general, it was not possible to ascertain which wolf produced each scat. However, we collected one or two scats per day depending on availability. When we collected two, we selected scats clearly apart or different, presumably from different times or individuals. We collected scats using sterile gloves, stored them individually into sterile 50 ml Falcon tubes with 100% ethanol and kept them at − 20 °C in the zoo. After collecting all samples, we transferred them to the lab, where we stored them at − 80 °C until analysis. Overall, we collected 50 scats.

### DNA extraction and sequencing

DNA extraction was performed using the QIAamp Fast DNA Stool Mini Kit (50) from QIAGEN (Ref. 51604). DNA amplification was performed by PCR using the 12SV05 marker^52^ of the 12 S gene, with primers 5′-TAGAACAGGCTCCTCTAG-3′ (forward) and 3′-TTAGATACCCCACTATGC-5′ (reverse), at the Analytical Services (SGIker) of the University of the Basque Country (UPV/EHU). We used the wolf blocking primer described by Shi et al. (2021), which increases the efficiency of prey DNA amplification (5´-CTATGCTTAGCCCTAAACATAGATAATTTTACAACAAAATAATTC/3SpC3/-3´). The amplification was carried out using 2 µL of DNA in a reaction mix containing 7.2 µL H20, 10ul Qiagen 2x and 0.40 µL of Fw and Rv primers (10µM). When blocking primer was included (10µM), the same amount of water was removed. We used blocking volumes equivalent to 5×, 10×, 15×and 20×the primer volume (2 µl, 4 µl, 6 µl and 8 µl respectively; hereafter 5×, 10×, 15× and 20× blocking primer concentration). We used blocking primer concentrations up to 20× to cover the ranges reported in previous works^32^. In a first analysis we included all the 50 samples without wolf blocking primer. Based on the results, we selected a subset of samples for further analysis with different blocking primer concentrations to determine its effect on the results. In this second run we included a subset of 30 samples with 5× and 10× blocking primer concentration, and a further subset of 25 samples with 15× and 20×. We selected the subsets based on the results of the first analysis, mainly number of diet reads in each scat, while maintaining a balanced representation of samples among diets. The thermocycler conditions were as follows: 95 °C for 15 min, 30 cycles of 94 °C for 30 s and 55 °C for 90 s (an elongation step was not included), 72 °C for 10 min, and 4 °C on hold. The samples were purified, and a second reaction was performed to index each amplified product and attach Illumina adaptors using the Illumina Nextera v2 kit. The PCR products were sequenced on an Illumina MiSeq NGS platform with a MiSeq v2 reagent kit of 300 cycles, according to the manufacturer’s instructions. We used blanks during DNA extractions (two blanks), which were amplified by PCR too. Moreover, we also included a PCR blank in the library constructs in each of the runs (one without blocking primer and another one with 10× blocking primer).

### Mock samples

To better understand the technical biases of the metabarcoding analysis, we constructed two types of mock samples. First (Mock type 1), we extracted DNA from the meats included in the diet, diluted the extracted DNA from each meat to 20 µg/ml and mixed this DNA at concentrations equal to those present in the diets. These mock samples were designed to evaluate the effect of sequencing. Secondly (Mock type 2), we prepared 0.2 g meat mixtures with the same proportions of meats as those offered in the wolf diets (Table 4) and then extracted DNA from those mixtures. These mock samples were created to assess the effect of the DNA extraction process. We performed DNA extractions using the DNeasy Blood & Tissue Kit (250) from QIAGEN (Ref. 69506). We generated two mock samples of each type for each diet, resulting in 12 mock samples of type 1 (2 replicates x 6 diets) and 12 mock samples of type 2. These simulated samples were processed in the same way as the fecal samples, although amplification was conducted at Universidad Autónoma de Madrid and sequencing at the sequencing service of the University Complutense de Madrid. We kept separate the analysis of feces and mock samples to avoid potential contamination.

### Data processing

We filtered obtained sequences for quality and trimmed them based on barcode length of 95 bp. Then, we merged the forward and reverse sequences and constructed a table of Amplicon Sequence Variants (ASVs), from which we removed chimeric sequences. Finally, taxonomy was assigned using a minBoot of 30 and the MIDORI database^53^ for 12 S gene, excluding most fish sequences (keeping only two for reference). This means that the algorithm performed the identification of each sequence 100 times, considering only those identifications that agreed at least 30 times as correct. We only considered those ASVs identified at the genus level (172 of 266, the 65%).

Once the sequences were assigned to genera, we removed all singletons and doubletons, as well as those samples with fewer than 100 reads of food items. Next, we grouped ASVs by genus, obtaining a read count per genus present in each scat. Finally, we estimated the relative read abundance (RRA) within prey DNA as the percentage of reads for each taxon relative to the total diet reads. In this study we used RRA as an approximation to volumetric method to assess diet.

### Statistical analyses

To achieve our goals, we conducted statistical analyses to address the following: (1) whether the use of a blocking primer increased the RRA of diet items and in what manner; (2) determine the number of item diets detected with DNA metabarcoding and whether it is affected by the use of a blocking primer; (3) approximate the time window of diet represented in each scat and whether it is affected by the blocking primer; (4) determine the accuracy of DNA metabarcoding in representing actual volumetric diet and whether this accuracy was affected by the blocking primer; and (5) how the accuracy of DNA metabarcoding varied with sample size, assessed through resampling techniques.

To evaluate whether and how blocking primer concentration influenced the representation of diet items in results, we performed a Generalized Linear Mixed Model (GLMM) with a Gaussian error and the Identity link function. In this model, RRA was the response variable, blocking primer concentration was the predictor, and scat identity was included as random factor.

To determine the number of item diets detected through DNA metabarcoding and assess the effects of the blocking primer in results, we tested for differences in numbers of food items detected in each scat without the blocking primer and with increasing concentrations of it. To assess the effect of blocking primer concentration on the number of items detected, we used proportional Generalized Lineal Models (GLMs) with a binomial error distribution and a logit link function^54^. In the proportional models, we used a combined response variable, treating each scat as a series of repeated binomial trials. For instance, in a scat from a diet with five items, where four were detected through metabarcoding, the data were modeled as five binomial trials (five items), four successful (detected items) and one failed (undetected item). The only predictor covariate was blocking primer concentration, treated as a factor with the “no blocking” condition as the reference category. To test for differences in the number of daily markers, we used a negative binomial GLM, with the number of markers detected as the response variable and blocking primer concentration as a categorical predictor.

To estimate the time window represented in scats and assess whether it was influenced by the use of a blocking primer, we determined the longest time period reflected in each sample. Specifically, we estimated that period by counting the number of days elapsed between the feeding of detected markers and the collection of the scat. Next, we performed a GLM with a Gaussian error distribution, using blocking primer concentration as predictor and the number of days as the response variable. Finally, we evaluated whether the use of a blocking primer led to a higher detection rate of wrong items –defined as items not part of the wolves’ diet before the scat was collected. Wrong items included diet species that were not part of the specific diet fed that week and daily markers that had not yet been consumed before scat production. To analyze that, we used a negative binomial GLM, with the number of wrong items detected in each sample as the response variable and blocking primer concentration as categorical predictor.

To assess the accuracy of DNA metabarcoding in representing volumetric diet composition, we tested the association between food composition and RRA of diet items. We used GLMMs with a Gaussian error distribution and the identity link function. We performed a model for each dataset: without the blocking primer and with 5×, 10×, 15× and 20× blocking primer concentrations. In these models, the RRA of each food item in each scat was the response variable and its percentage in the diet as the predictor. We included food type (species) and scat ID as crossed random effects. If reads of non-fed species were detected, we incorporated them into the regressions by assigning the RRA value to the response variable and a zero value as the predictor for their percentage in the diet. To evaluate the representativeness of this semi-quantitative approach, we calculated the marginal (*R*^2^_*marg*_) and conditional (*R*^2^_*con*_) coefficients of determination well^55^. The marginal *R*^2^ represents the proportion of variance explained by the fixed factors of the GLMM, indication how well metabarcoding results approximated the actual diet. The conditional *R*^2^ accounts for both fixed and random effects, incorporating variations due to individual scats or specific food items. To identify potential extraction or primer biases, we analyzed mock samples in the same way as DNA extracted from scats. We ran a GLMM with Gaussian error distribution, using the percentage of reads of each species as the response variable and its percentage in the mock composition as the predictor. To account for species-specific variation, food type (species) was included as a random effect. Since we used two types of mock samples –one mixed from extracted DNA and another from DNA extracted directly from meat mixtures– we added mock type as a factor in the model to determine whether separate models were necessary.

To assess how the accuracy of DNA metabarcoding varied with sample size, we examined its effect on the coefficient of determination. To simplify the results, we conducted this analysis only for the blocking primer concentration that yielded the highest coefficient of determination. We randomly selected scats in different sample sizes (ranging from 20 to 50, increasing by one at each step), re-ran the model, and extracted the coefficient of determination. In the resampling process, to keep results realistic, we also included those scats that did not produce enough DNA for analyses. Additionally, we did not simulate sample-sizes beyond 50 to keep simulations within the range of actual samples. We repeated this procedure 1000 times for each sample-size assessed. Finally, we averaged the coefficients of determination across the 1000 resamples and inspected their dispersion.

We performed all analyses using R v4.0.4^56^. For bioinformatic analyses we used Cutadapt software for primer removal^57^ and the DADA2 v1.22.0 for subsequent processes, including filtering, assembling and taxonomic identification^58,59^. For GLMMs we used the package lme4 v1.1-19^60^. To calculate *R*^2^ of GLMMs, we employed PiecewiseSEM v1.2.1^61^, and we generated plots with ggplot2 v3.1.0^62^ package. Unless stated otherwise, values reported in descriptive statistics are the mean ± one standard deviation with the range provided in parentheses.

## Supplementary Information

Below is the link to the electronic supplementary material.

Supplementary Material 1

## Data Availability

The raw Illumina sequences generated and analysed during the current study are available in the European Nucleotide Archive (ENA) repository (https://www.ebi.ac.uk/ena/browser/home) under the accession number PRJEB92094 (secondary study accession: ERP175021).
