# Supplementary material for: Metabarcoding provides accurate estimation of volumetric diet composition in a top predator despite interference from blocking primers
Source: Sci Rep. 2025 Aug 8;15:29033. doi: 10.1038/s41598-025-14837-9 (PMC12334639; doi:10.1038/s41598-025-14837-9)

Supplementary material for:

**Metabarcoding provides accurate estimation of volumetric diet composition in a top predator despite interference from blocking primers**

Table S1: Species added in small quantities (<1%) to meatballs as time markers. “Day” indicates in which day they were included, the order being the same in all diets. We provide common English and Latin names of species.

| Day | Marker Speceis | |
| --- | --- | --- |
| 1 | Goat | *Capra hircus* |
| 2 | Water buffalo | *Bubalus bubalis* |
| 3 | Reindeer | *Rangifer tarandus* |
| 4 | Guanaco | *Lama guanicoe* |
| 5 | Quail | *Coturnix coturnix* |
| 6 | Zebra | *Equus quagga* |
| 7 | Mallard | *Anas platyrhyncos* |

**Analysis of extraction bias or primer bias**

We found no evidence of extraction bias or primer bias. This was shown by the significant association and good fit (*R^2^_marg_* = 0.878, Table S2; Fig. S1) between percentage of DNA or meat in the mixture of mock samples and the relative read abundance obtained from mocks. Thus, the number of reads of each species obtained was closely associated to the amount of its DNA in the sample and, therefore, no considerable primer bias. Further, we found no evidence for effects of mock types, mixture of DNAs extracted and mixture of meats and common extraction (Table 6), suggesting no extraction bias and also that the different meat types contained similar amounts of DNA per mass unit.

Table S2: Results of the GLMM of the association between mock sample composition fed and proportions of reads obtained. For random variables we show their variance (Var) and standard error (SE) associated with them. For fixed factors we show the estimate (Est) of the slope and its standard error (SE), the value of the t statistic (t val), and the associated P value (*P*). We also indicate the sample size (N) of the model and its marginal (*R^2^_marg_*) and conditional (*R^2^_con_*) coefficient of determination. Mock type refers to mocks created mixing extracted DNA or those created by extracting DNA from a mixture of meats.

|  | Variable | | |  |  |  |  |  |
| --- | --- | --- | --- | --- | --- | --- | --- | --- |
| **No blocking** | Fixed | | Random | Var | Est | SE | t val | *P* |
| N= 216 |  | Species (N=9) | | 43.18 |  | 6.571 |  |  |
| *R^2^_marg_*=0.878 | Intercept | |  |  | 0.519 | 1.180 | 0.440 | 0.667 |
| *R^2^_cond_*=0.898 | % in mock | |  |  | 0.953 | 0.024 | 39.763 | <0.001 |
|  | Mock type | |  |  | -0.002 | 0.894 | -0.002 | 0.998 |

Table S3: Results of the negative binomial GLM assessing differences in the number of wrong items detected and the concentration of blocking primers as categorical factor. For each blocking primer concentration used, the intercept being no blocking primer, we show: the regression estimate (Est) and its standard error (SE), the value of the t statistic (t val), and the associated P value (*P*). We also show the average number of wrong items (Avg. wrong items) per sample with its standard deviation within parenthesis and the sample size (N)

| Blocking primer concentration | Est | SE | t val | *P* | Avg. wrong items | N |
| --- | --- | --- | --- | --- | --- | --- |
| Intercept (0x) | 2.191 | 0.256 | 8.567 | <0.001 | 0.50 (± 0.96) | 34 |
| 5x | -0.648 | 0.323 | -2.004 | 0.045 | 1.11 (± 0.99) | 28 |
| 10x | -0.789 | 0.321 | -2.458 | 0.014 | 1.18 (± 1.28) | 28 |
| 15x | -0.838 | 0.328 | -2.559 | 0.011 | 1.30 (± 1.29) | 23 |
| 20x | -0.813 | 0.332 | -2.451 | 0.014 | 1.22 (± 1.17) | 23 |

Figure S1: Association between composition of mock samples and percentage of reads obtained without blocking primer. Points represent proportion of each species in mock samples. The thick blue line indicates the average trend produced by the GLMM with diet species as random effect. Thinner lines show linear regression fits over predicted values for different diet species included in the study.


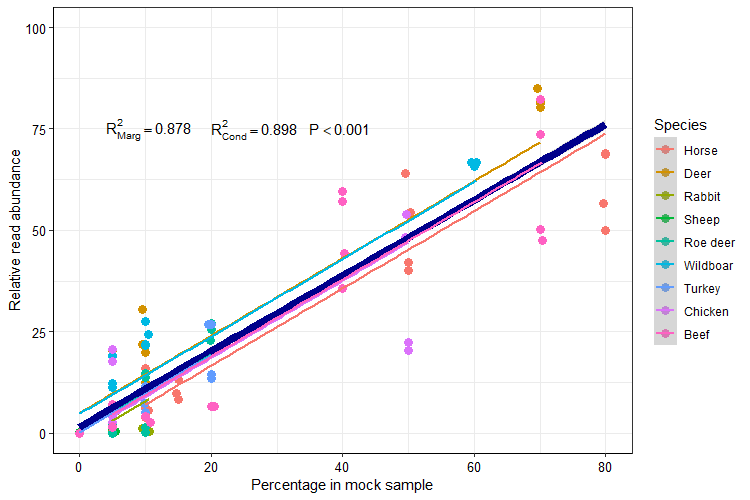


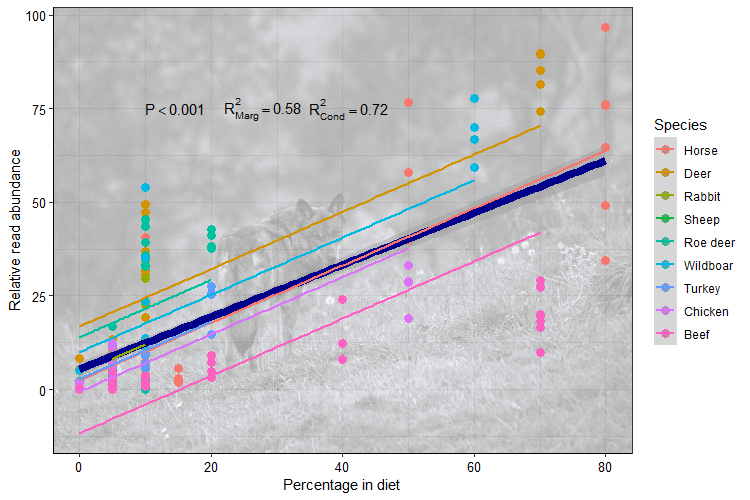
Figure S2: Association between composition of diet fed and percentage of diet reads with 5x blocking primer concentration. Points represent proportion of each diet item in samples. The thick blue line indicates the average trend produced by the GLMM with diet species as random effect. Thinner lines show linear regression fits over predicted values for different diet species included in the study.

Figure S3: Association between composition of diet fed and percentage of diet with 10x blocking primer concentration. Points represent proportion of each diet item in samples. The thick blue line indicates the average trend produced by the GLMM with diet species as random effect. Thinner lines show linear regression fits over predicted values for different diet species included in the study.


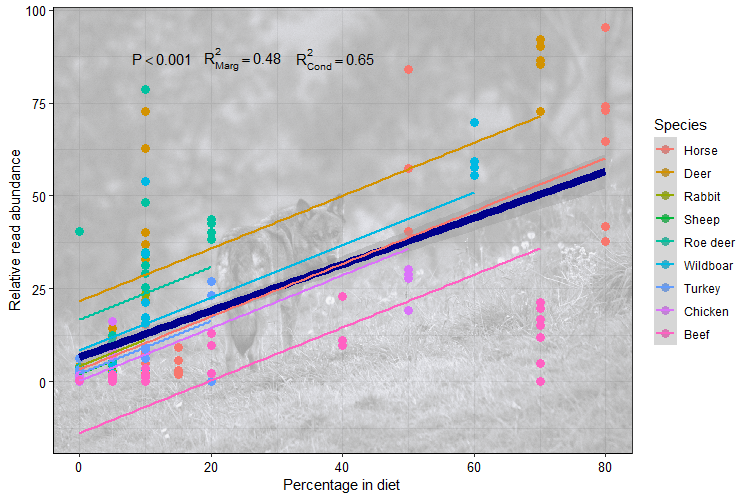


Figure S4: Association between composition of diet fed and percentage of diet with 15x blocking primer concentration. Points represent proportion of each diet item in samples. The thick blue line indicates the average trend produced by the GLMM with diet species as random effect. Thinner lines show linear regression fits over predicted values for different diet species included in the study.


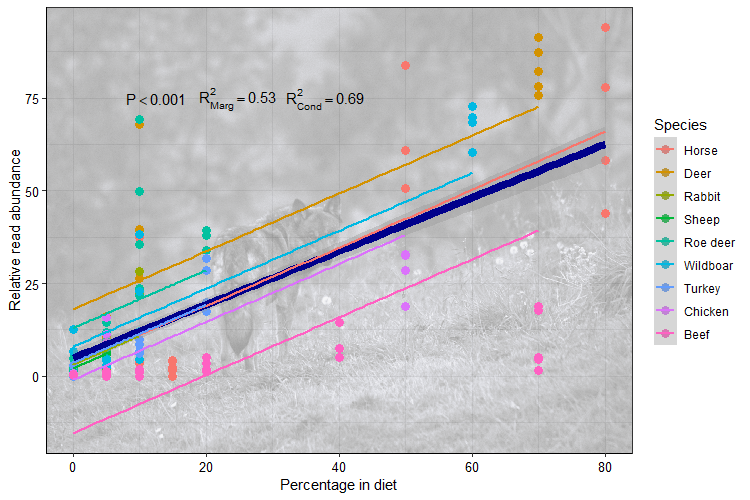


Figure S5: Association between composition of diet fed and percentage of diet with 20x blocking primer concentration. Points represent proportion of each diet item in samples. The thick blue line indicates the average trend produced by the GLMM with diet species as random effect. Thinner lines show linear regression fits over predicted values for different diet species included in the study.


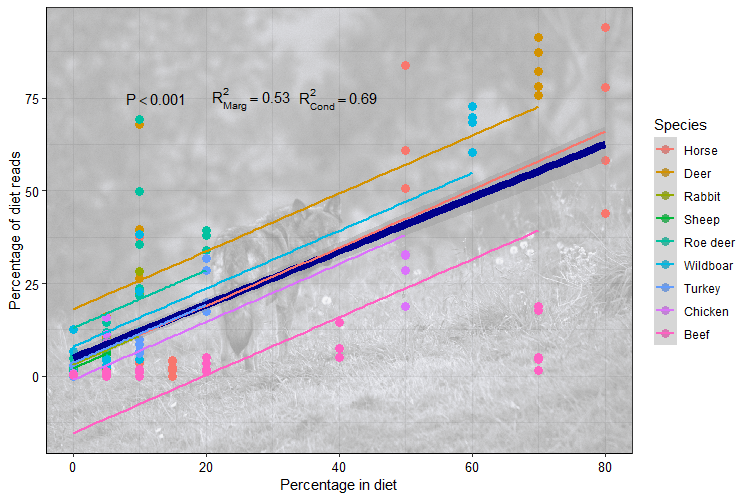

Supplement: Supplementary file 1 — Supplementary Material 1 [file 41598_2025_14837_MOESM1_ESM.docx]
